# Prevalence of human papilloma virus (HPV) and its genotypes in cervical specimens of Egyptian women by linear array HPV genotyping test

**DOI:** 10.1186/s13027-016-0053-1

**Published:** 2016-02-17

**Authors:** Mohamed A. Youssef, Lobna Abdelsalam, Reem Abdelhameed Harfoush, Iman Mamdouh Talaat, Eman Elkattan, Abeer Mohey, Rana M. A. Abdella, Marwa Salah Farhan, Hany Ahmed Foad, Abeer Mostafa Elsayed, Naglaa A. Elkinaai, Doaa Ghaith, Mohamed Elsayed Rashed, Mohamed Abd-El Ghafar, Yasser Khamis, Ahmed N. Hosni

**Affiliations:** Department of obstetrics& gynecology, Faculty of Medicine, Cairo University, Cairo, Egypt; Department of clinical & chemical pathology, Faculty of medicine, Cairo University, Cairo, Egypt; Department of medical microbiology & immunology, Faculty of medicine, Alexandria University, Alexandria, Egypt; Department of Pathology, Faculty of Medicine, Alexandria University, Alexandria, Egypt; Department of chemical pathology, Faculty of Medicine, Cairo University, Cairo, Egypt; Department of clinical pathology, Faculty of Medicine, Cairo University, Cairo, Egypt; Department of Pathology, National Cancer Institute, Cairo University, Cairo, Egypt; National Organization for Research & Control of Biologicals (NORCB), Cairo, Egypt; Department of Obstetrics& Gynecology, Beni-Suef University, Beni Suef, Egypt; Egyptian International Fertility IVF-ET center-Cairo, 16 Elhassan Ben Ali, Nast City, Cairo Egypt

**Keywords:** Human papillomavirus (HPV), Cervical smear, Linear Array HPV genotyping, Cervical squamous intraepithelial lesions

## Abstract

**Background:**

The association of human papillomavirus (HPV) with cervical cancer is well established.

**Aim:**

To investigate HPV genotype distribution and co-infection occurrence in cervical specimens from a group of Egyptian women.

**Methods:**

A group of 152 women with and without cervical lesions were studied. All women had cervical cytology and HPV testing. They were classified according to cytology into those with normal cytology, with squamous intraepithelial lesions (SIL) and invasive squamous cell carcinoma (SCC). Cervical samples were analyzed to identify the presence of HPV by PCR, and all positive HPV-DNA samples underwent viral genotype analysis by means of LINEAR ARRAY HPV Genotyping assay.

**Results:**

A total of 26 HPV types with a prevalence of 40.8 % were detected. This prevalence was distributed as follows: 17.7 % among cytologically normal females, 56.5, 3.2, and 22.6 % among those with LSIL, HSIL and invasive SCC respectively. Low-risk HPV types were detected in 81.8 % of the cytologically-normal women, in 5.7 % of those in LSIL women, and in 14.3 % of infections with invasive SCC, while no low-risk types were detected in HSIL. High-risk HPV types were detected in 18.2 % of infections in the cytologically normal women, 14.3 % of infections in LSIL, and in 21.4 % of invasive lesions. The probable and possible carcinogenic HPV were not detected as single infections. Mixed infection was present in 80 % of women with LSIL, in 100 % of those with HSIL, and in 64.3 % of those with invasive SCC. This difference was statistically significant. HPV 16, 18 and 31 were the most prevalent HR HPV types, constituting 41.9, 29.03 and 12.9 % respectively, and HPV 6, 62 and CP6108 were the most prevalent LR HPV types constituting 11.3, 9.7 and 9.7 % respectively.

**Conclusion:**

These data expand the knowledge concerning HPV prevalence and type distribution in Egypt which may help to create a national HPV prevention program. HPV testing using the LINEAR ARRAY HPV Genotyping assay is a useful tool when combined with cytology in the diagnosis of mixed and non-conventional HPV viral types.

## Background

With 528 000 new cases every year, cervical cancer is the fourth most common cancer affecting women worldwide [1]. Almost 70 % of the global burden falls in areas with lower levels of development [2]. Persistent infection with human papillomavirus (HPV) is the principal cause of cervical cancer and its precursor cervical intraepithelial neoplasia (CIN) [3].

Data is not yet available on the HPV burden in the general population of Egypt. Current estimates indicate that cervical cancer ranks as the 13th most frequent cancer among women in Egypt and the 10th most frequent cancer among women between 15 and 44 years of age [4]. 

About 40 HPV types can infect the genital mucosa. However, only a restricted group of HPVs, termed high-risk (HR) HPVs, are associated with high-grade cervical precancer lesions (CIN) or cervical cancer, while the low-risk (LR) types are generally associated with condylomas or other benign epithelial lesions [5].

HR-HPV 16 and 18 are estimated responsible for nearly 70 % of cervical cancer cases worldwide [6]. In Northern Africa, the region Egypt belongs to, about 3.0 % of women in the general population are estimated to harbor cervical HPV-16/18 infection at a given time and 81.2 % of invasive cervical cancers are attributed to HPVs 16 or 18 [4].

Development of cellular dysplastic changes is influenced by numerous factors, such as smoking habit, immune suppression, oral contraceptive use, hormone replacement therapy as well as host genetic makeups [7–9]. Progression of the initial cytopathologic process to micro invasive and invasive cancer usually takes decades [10]. 

Two types of prophylactic vaccines have been developed to prevent cervical cancer: a bivalent vaccine against HR-HPV 16 and 18, and a tetravalent vaccine against HR-HPV 16/18 and LR-HPV 6 and 11 [10–12].

Some studies suggest that these vaccines seem to protect against some HPV 16-related types (31, 33, 35 and 52) and HPV 18-related types (39, 45, 59, 68 and 85) [13].

Cervical cancer prevention programs in both developing and developed nations have generally relied on cytological testing using the conventional Papanicolaou’s (Pap) test [14, 15]. However, cervical cytology suffers from relatively high false-negative rates (low sensitivity) as well as high false-positive rates (low specificity) [15].

The presence of HPV episomal DNA in the epithelial cells is often associated with koilocytotic atypical changes as the manifestation of HPV-induced cytopathology in cervical dysplasia, a reversible early precancerous pathology which can be recognized on Pap smears and in histopathologic sections as low-grade cervical intraepithelial neoplasia (CIN1), the precursor stage in the development of a CIN2 and a CIN3 lesion [16]. This link between a virology finding and cytopathic changes in cervical carcinogenesis has led to the proposed guidelines of using HPV DNA testing to replace the traditional Pap smear cytology as the primary screening for referral to colposcopy of HPV-16/HPV-18-positive women with negative cytology for cervical cancer prevention [17, 18].

The HPV DNA test used to replace the traditional Pap cytology screening for precancerous cells and cancer cells in the cervico-vaginal samples must be highly sensitive, capable of detecting not only the HPV DNA with high copy number in the koilocytes of the low grade CIN lesions, but also the HPV DNA in the cervical cancers which may have as low as one single copy of HPV DNA per cancer cell, and to generate a reliable genotyping result of the detected HPV. Most commercially available HPV test kits have been shown to be suboptimal in analytical sensitivity and typing specificity [19].

This study main objective was to assess the prevalence of HPV genotypes in Egyptian women, attending routine gynecological examinations in different age groups, with and without pre-invasive and invasive cervical lesions and its correlation to findings in cytology. The study also provides useful information on the performance and clinical validity of HPV screening using the LINEAR ARRAY HPV Genotyping test.

## Methods

### Study design and population

This multicenter, cross sectional study included 152 female subjects seeking routine gynecologic care at the family planning and gynecological outpatient clinic, Al Kasr Al Aini University Hospital, Cairo, and the outpatient clinics of the Obstetrics and Gynecology Department at El Shatby Maternity Hospital, Alexandria University, during the period from January 2013 through May 2014. The inclusion criteria for patient selection included age 20 years and over, chronic vaginal infection, post-coital bleeding, irregular menstruation, and lower-back pains. The objective of the study was explained and written consent was obtained from each participant according to the ethics committee guidelines.

### Ethical considerations

The study was conducted in accordance with the Declaration of Helsinki and the protocol of the study was approved by the Faculty of Medicine, Alexandria University Ethics Committee prior to its start.

### The participants were subjected to:

1. Complete history taking: Residency (rural or urban), occupation, smoking, contraception, and reproductive history (parity)
2. Cytology sample: Cervical cell scrapings were collected by a gynecologist with a cytobrush from the ecto- and endo-cervix of the uterus. A slide was prepared for conventional Pap cytology and the cytobrush was then placed in specimen transport medium and transported in PreservCyt Solution (Roche diagnostics, USA). Samples were stored at –80 °C until HPV molecular analysis. Cytological findings were classified in line with the 2004 Bethesda classification system [14], as follows: (1) within normal limits or reactive cellular changes (normal), (2) atypical squamous cells (a) low-grade squamous intraepithelial lesion (LSIL), and (b) high-grade squamous intraepithelial lesion (HSIL).
3. Colposcopy: for suspicious lesions.
4. Tissue biopsies were taken from suspicious lesions on colposcopic examination. The histologic diagnosis of the squamous lesions was classified according to the WHO nomenclature and criteria as follows [16]; Cervical Intraepithelial Neoplasia (CIN) grade 1 (CIN 1), grade 2 (CIN 2), grade 3 (CIN 3), and invasive SCC.
5. HPV detection
  i. DNA extraction: from cervical samples using (AmpliLute Liquid Media Extraction Kit), according to the manufacturer’s instructions. Extracted HPV and human genomic DNA were immediately stored at−20° C until PCR amplification.
  ii. Screening PCR [20]: for the detection of HPV DNA presence in the cervical samples, before proceeding to genotyping, as follows:
    β-Globin gene PCR: DNA integrity was assessed by PCR amplification of a 268 bp segment of the human β-Globin gene using the following primers: forward; 5′-ACACAACTGTGTTCACTAGC-3′; and reverse; 5′-CAACTTCATCCACGTTCACC-3′, as previously described.
    HPV PCR: β-Globin positive samples were subjected to PCR that amplifies 450 bp product for L1 open reading frame (ORF) of HPV, using forward primer; 5′-TTTGTTACTGTGGTAGATACTAC-3′ and reverse primer; 5′-AAAAATAAACTGTAAATCATATTC-3′ as previously described. Each run included an HPV positive control and a negative (water) control. The amplification products were visualized by means of electrophoresis analysis on 2 % agarose gels containing ethidium bromide (0.5 mg/ml).
  iii. HPV genotyping:
    HPV positive samples were genotyped using the LINEAR ARRAY HPV Genotyping test (LA): (Roche Diagnostics, Indianapolis, IN, USA).
    This technique is based on PCR amplification of target DNA using L1 consensus HPV primer PGMY09/11, hybridization of the amplified product to oligonucleotide probes and their detection by colorimetric reaction. Particularly, the master mix contains primers for the amplification of a 450-bp fragment of the L1 region of more than 37 HPV genotypes and a 268-bp fragment of the human β- globin gene. The detection and genotype determination was performed using the denatured amplified DNA and an array of oligonucleotide probes, located in the polymorphic region of L1 that permitted independent identification of individual HPV genotypes. Negative samples were included in each assay to demonstrate the specificity of the test. The HPV types detected include 12 HR-HPV genotypes (HPV16, 18,31, 33, 35, 39, 45, 51, 52, 56, 58, 59), 1 probably carcinogenic (HPV 68), 13 LR-HPV genotypes (HPV6, 11, 40, 42, 54, 55, 61,62,72, 81,83, 84 and CP6108), 7 possibly carcinogenic genotypes (HPV 26, 66, 53, 67, 70, 73, 82) and 4 genotypes for which the risk is still undetermined (HPV64, 69, 71, and IS39)]

### Statistical analysis

Data were fed to the computer and analyzed using IBM SPSS software package version 20.0. Qualitative data were described using frequency and percentage. Quantitative data were described using range (minimum and maximum), mean, and standard deviation. Comparison between different groups regarding categorical variables was tested using Chi-square test. When more than 20 % of the cells have expected count less than 5, correction for chi-square was conducted using Yate’s correction (linear-by-linear association) for 2x2 tables or Monte Carlo Correction for >2x2 tables. Comparison between two independent populations was done using independent samples *t*-test. Significance of the obtained results was judged at the 5 % level.

## Results

**Fig. 1:**
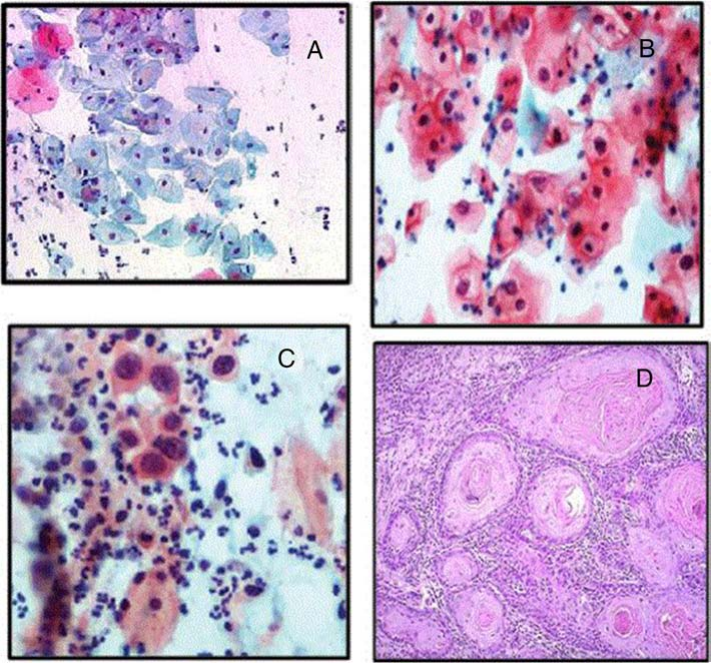
**a** Cytologic features of normal squamous epithelial cells with small pyknotic nuclei (Pap, x200). **b** Low-grade squamous intraepithelial lesion with Human Papilloma Viral changes (Pap, x400). **c** High-grade squamous intraepithelial lesion. Nuclei are greatly enlarged, vary in size and shape, and contain hyperchromatic, coarsely granular clumped chromatin (Pap, x400). **d** Invasive SCC showing nests of neoplastic squamous cells invading through a chronically inflamed stroma (H&E, x200)

One hundred fifty two female subjects seeking routine gynecologic care at the family planning and gynecological outpatient clinic, Al Kasr Al Aini University Hospital, Cairo, and the outpatient clinics of the Obstetrics and Gynecology Department at El Shatby Maternity Hospital, Alexandria University, were recruited. Table 1 shows the demographic, clinical and laboratory characteristics of the studied group. Regarding the cyto and histo-pathological findings of the studied specimens, the cases were categorized into normal (Fig. 1a), LSIL (Fig. 1b), HSIL (Fig. 1c) and invasive SCC (Fig. 1d) in 67.1, 21.7, 2.0, and 9.2 % respectively. The percentage of HPV positivity in the studied specimens was 40.8 % (62/152).

**Table 1:** Demographic characteristics, clinical and laboratory findings of studied group

Characteristics	(*N* = 150 (%)
Age (years) Mean ± SD	29.69 ± 4.88
Parity	
Nullipara	25 (16.4 %)
Multipara	127 (83.6 %)
Residence	
Urban	104 (68.4 %)
Rural	48 (31.6 %)
Occupation	
Housewife	116 (76.3 %)
Worker	36 (23.7 %)
Smoking Status	
No	136 (89.5 %)
Yes	16 (10.5 %)
Contraception method	
None	62 (40.8 %)
IUCDs^a^	16 (10.5)
Injectables	10 (6.6 %)
COCPs^b^	36 (23.7 %)
POPs^c^	13 (8.6 %)
Condoms	15 (9.9 %)
Gynaecological findings	
Normal	97(63.8 %)
Multiple genital warts	12 (7.9 %)
Intermenstrual bleeding	13 (8.5 %)
Post-coital bleeding	30 (19.7 %)
Pap smear & biopsy results	
Normal	101 (67.1 %)
LSIL^d^	35 (21.7 %)
HSIL^e^	2 (2.0 %)
Invasive SCC^f^	14 (9.2 %)
HPV DNA	
Negative	90 (59.2 %)
Positive	62 (40.8 %)

^a^ *IUCD* intra uterine contraception device; ^b^ *COCPS* Combined oral contraceptive pills; ^c^ *POPS* Progesterone only pills; ^d^ *LSIL* low grade squamous intra-epithelial lesion; ^e^ *HSIL* high grade squamous intra-epithelial lesion; ^f^ *SCC* squamous cell carcinoma

**Fig. 2:**
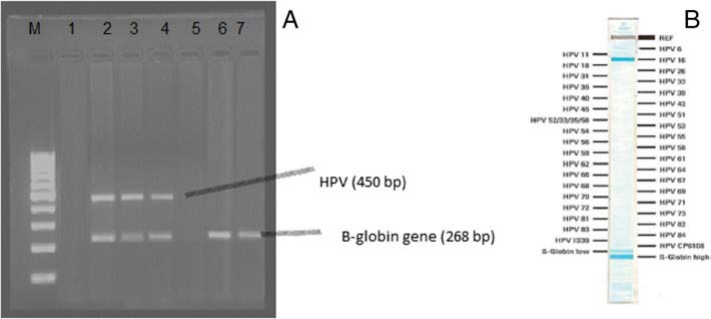
**a** 2 % agarose gel electrophoresis showing 100 molecular weight marker (M), positive samples (HPV and β-globin genes DNA) in lanes 2, 3, 4, negative samples (β-globin genes DNA only) in lanes 6 & 7 and negative controls (lanes 1 & 5). **b** Linear Array HPV genotyping test strip showing a colored band corresponding to HPV16 and β-globin genes

Table 2 investigates the relation between the risk factors and HPV infection. It was shown that HPV positive cases were mainly in age groups between 26–30 years and 31–35 years (30.6 %), and this distribution was statistically insignificant (*p* = 0.741). The reproductive history, residence, occupation, smoking habit, use of contraception and history of irregular genital bleeding of the studied group showed a statistically insignificant difference between HPV negative and positive cases. The only finding that showed statistical significance in relation to HPV status was the presence of genital warts (*P* = 0.000). Table 2 shows also the distribution of HPV DNA status according to cervical cytology and biopsy results. All HPV negative cases showed normal cytology, while 82.3 % of the HPV positive cases showed cytological changes: 56.5 % with LSIL, 3.2 % with HSIL and 22.6 % with invasive SCC. The % raw results showed that 10.9 % of the normal cytology results were HPV positive, while all abnormal cytology and biopsy samples were HPV positive. This distribution was statistically significant (*P* = 0.000).

**Table 2:** Relation and distribution of studied risk factors to HPV DNA status

	DNA results (*n* = 152)		*p* value
	Negative (*n* = 90)	Positive (*n* = 62)	
Age (years) Mean ± S.D.	29.53 ± 4.61	29.93 ± 5.27	0.619
Age group (n, %)			
*21-25 years*	19 (21.1 %)	14 (22.6 %)	
*26-30 years*	35 (38.9 %)	19 (30.6 %)	0.741
*31-35 years*	25 (27.8 %)	19 (30.6 %)	
*35-40 years*	11 (12.2 %)	10 (16.1 %)	
Parity			
*Nullipara*	15 (16.7 %)	10 (16.1 %)	0.930
*Multipara*	75 (83.3 %)	52 (83.9 %)	
Residence			
*Urban*	62 (68.9 %)	42 (67.7 %)	0.881
*Rural*	28 (31.1 %)	20 (32.3 %)	
Occupation			
*Housewife*	70 (77.8 %)	46 (74.2 %)	0.609
*Worker*	20 (22.2 %)	16 (25.8 %)	
Smoking			
*No*	81 (90.0 %)	55 (88.7 %)	0.799
*Yes*	9 (10.0 %)	7 (11.3 %)	
Contraception			
*No*	39 (43.3 %)	23 (37.1 %)	0.442
*Yes*	51 (56.7 %)	39 (62.9 %)	
Gynecological findings			
*Normal (n = 97)*	65	32	
*Multiple genital warts (n = 12)*	0.0 (0 %)	12 (19.4 %)	0.000
Intermenstrual bleeding (13)	8.0 (8.9 %)	5.0 (8.1 %)	0.858
Post-coital bleeding (30)	17 (18.9 %)	13 (21.0 %)	0.752
Distribution of HPV DNA status according to cervical cytology and biopsy results			
*Normal (n = 101)*	90 (100)	11 (17.7)	0.000^a^
*LSIL (n = 35)*	0.0	35 (56.5)	
*HSIL (n = 2)*	0.0	2.0 (3.2)	
*Invasive SCC (n = 14)*	0.0	14 (22.6)	

^a^ *MC* Monte Carlo Sig. (2-sided)

Table 3 demonstrates that 23 women were found to be infected by a single HPV type (10 HR and 13 LR types) while 39 women showed co-infection. The prevalence of HPV-positive cases among normal Pap smears was 17.7 % (11/62), whereas the prevalence among those with LSIL, HSIL and invasive SCC was 56.5 % (35/62), 3.2 % (2/62), and 22.6 % (14/62), respectively. LR HPV types were detected in 81.8 % (9/11) of the infections in cytologically normal women, in 5.7 % (2/35) in women presenting with LSIL and in 14.3 % (2/14) of infections diagnosed with invasive SCC, while no LR types were detected in HSIL. HR HPV types were detected in 18.2 % (2/11) of infections in cytologically normal women, in 14.3 % (5/35) of infections in LSIL, and in 21.4 % (3/14) invasive lesions. The probable and possible carcinogenic HPV were not detected as single infections. HPV DNA was detected as mixed infection in 80 % (28/35) of women with LSIL, in 100 % (2/2) of those with HSIL, and in 64.3 % (9/14) of those with invasive SCC. The difference in the distribution was statistically significant (*p* = 0.000).

**Table 3:** Frequency of HPV positivity according to the type of cervical lesion found

Pathological diagnosis	HPV DNA (62)			
	Total (n, %)^a^ HPV + ve cases	(N, %)^b^ single HPV cases		(N, %)^b^ multiple HPV cases
		LR	HR	
Normal	11 (17.7)	9(81.8)	2(18.2)	0 (0.0)
LSIL	35 (56.5)	2 (5.7)	5 (14.3)	28 (80)
HSIL	2 (3.2)	0(0.0)	0 (0.0)	2 (100)
Invasive SCC	14 (22.6)	2(14.3)	3 (21.4)	9 (64.3)
Total	62 (100)	13(20.9)	10 (16.1)	39 (62.9)

*X* ^2^ = 33.683, p_MC_ = 0.000*

^a^Percentage referred to the total number of HPV positivity

^b^Percentage referred to the number of HPV positive corresponding lesion

Table 4 shows that a total of 26 different HPV types were detected in this study: 10 HR, 11 LR, 1 probably carcinogenic and 4 possibly carcinogenic. Type 16 is the most common type (41.9 %; 26/62), followed in order of decreasing frequency, by HPV 18 (18/62; 29.03 %), HPV 31 (8/62; 12.9 %), HPV 58, 59, 6 (7/62; 11.3 %), HPV 45, 62, CP6108 (6/62;9.7 %), HPV 84 (5/62;8.1 %), HPV 33, 51, 66 (4/62;6.5 %), HPV 35, 52, 11, 61, 68 (3/62;4.8 %), HPV 72, 81, 83 (2/62; 3.2 %) and HPV 40, 54, 67, 70, 73 (1/62; 1.6 %).

**Table 4:** HPV prevalence and type distribution

HPV prevalence/type	N, %		
HPV negative lesions	90/152 (59.2)		
HPV positive lesions	62/152 (40.8)		
	Single	Multiple	Total
	23/62 (37.1)	39/62 (62.9)	62 (100)
High risk HPV			
16	5 (8.6)	21(33.9)	26 (41.9)
18	3 (4.8)	15(24.2)	18 (29.03)
31	0(0.0)	8(12.9)	8 (12.9)
33	0(0.0)	4(6.5)	4 (6.5)
35	0(0.0)	3(4.8)	3(4.8)
45	0(0.0)	6 (9.7)	6(9.7)
51	1(1.6)	3(4.8)	4(6.5)
52	0(0.0)	3(4.8)	3(4.8)
58	1(1.6)	6(9.7)	7(11.3)
59	0(0.0)	7(11.3)	7(11.3)
Low risk HPV			
6	3(4.8)	4(6.5)	7(11.3)
11	2(3.2)	1(1.6)	3(4.8)
40	1(1.6)	0 (0.0)	1(1.6)
54	1(1.6)	0(0.0)	1(1.6)
61	0(0.0)	3(4.8)	3(4.8)
62	1(1.6)	5(8.6)	6(9.7)
72	0(0.0)	2(3.2)	2(3.2)
81	0(0.0)	2(3.2)	2(3.2)
83	0(0.0)	2(3.2)	2(3.2)
84	1(1.6)	4(6.5)	5(8.6)
CP6108	4(6.5)	2(3.2)	6(9.7)
Probably carcinogenic			
68	0(0.0)	3(4.8)	3(4.8)
Possibly carcinogenic			
66	0(0.0)	4(6.5)	4(6.5)
67	0(0.0)	1(1.6)	1(1.6)
70	0(0.0)	1(1.6)	1(1.6)
73	0(0.0)	1(1.6)	1(1.6)

## Discussion

This is the most recent study to estimate the prevalence and type distribution of HPV in Egyptian women using in vitro diagnostic (IVD) technique and by far it is the largest HPV genotypes tested in a single study. In our study, conducted at two large hospitals, 40.8 % (62/152) of women attending routine gynecological examinations were found to be HPV-positive. This was found to be higher than the overall prevalence reported previously in Egypt; 10.4 % [19], 10.3 % [4] and 15 % [15], in Northern Africa (10.9 %) [4], and globally (11.4 %) [19]. This might be attributed to the fact that this study was cross-sectional, and as HPV infections may be transient and resolve on their own, the prevalence of HPV might therefore change over time. Secondly, since the cervical samples were collected only from women attending routine gynecological visits, there is a possibility of selection bias. Nevertheless, a study in Egypt of HPV infection among a high-risk group of female patients using PCR in-situ hybridization of cervical biopsies revealed an approximately 70 % infection rate [15]. This high rate of infection is attributed to the promiscuous sexual behavior of the sampled women. Different rates of infection have been recorded in different parts of the world. Studies from different regions of the world such as Greece have reported an overall HPV prevalence ranging from 22.7 to 50.7 % [21, 22]. Kuhn et al. [23] in the USA, Sellors et al. [24] in Canada, and Sun et al. [25] in Taiwan reported HPV-positive rates of 18.1, 24, and 21 %, respectively. On the other hand, Varghese et al. [26] reported 6.1 % among Indian women. This augments the findings of Becker et al. [27] that there are regional and ethnic differences in HPV prevalence. 

While HPV infection is considered a necessary precursor to cervical cancer, it is not a sufficient cause. Other determinants of the progression of HPV infection to cervical cancer relate to the woman’s immune status. Those with an immune system compromised as a result of malnutrition, pregnancy, or immunosuppressive chemotherapy appear to be at increased risk of progression [28]. Additional cofactors, such a cigarette smoking, may be required as a carcinogenic to advance HPV-infected cells toward neoplastic progression [29]. Similarly, the study of Abdel Aziz M.T. et al. [15], parity, smoking, history of STD infection, as well as clinical vaginitis were among the significant risk predictors distinguishing HPV-negative and positive cases. On the other hand, smoking and parity were studied among other predisposing factors in the current study, and were found to be insignificantly associated with HPV. This might be due to our small sample size. The only finding that was statistically significant was the presence of genital warts.

HPV infection is frequent among young women. However, most of the infections are cleared over a period of 6–12 months and only a small percentage develops a persistent infection [5]. In the present study, there is a higher HPV prevalence at younger ages (26–35 years of age), but this was statistically insignificant. Several researchers have found HPV infection rates to be higher in younger age groups and it mainly depends on the age of starting sexual activity [1, 24, 30]. Religion and cultural upbringing of the Egyptian women may play a role in protecting them from earlier infection [15].

In our study, HPV DNA positivity was 10.9 % (11/101) in those with normal cytology. This agrees with the study of Argyri E et al. [31], where HPV prevalence in normal samples was 15.7 %. For Eastern Europe, HPV prevalence in a meta-analysis of 4053 samples tested with normal cytology was 21.4 % [32]. Similarly, the study of Ozturk and coworkers [1] reported that HPV DNA positivity was 2.1 % in women with normal cervical cytology and 42.9 % in women with epithelial cell abnormalities. They also reported that the presence of HPV together with high rates of epithelial cell abnormalities leading to cervical cancer supported the association between HPV and malignancy. Also, in the study of Abdel Aziz MT et al. [15], 28 % (7/106) of those who were classified as having normal cytology recorded positive HPV DNA. The study of Panatto and coworkers [33] showed that 18.2 % of the sexually active females with normal cervical cytology were infected by at least one HPV genotype. Ho and coworkers [29] reported that women who are HPV DNA positive and lack cervical lesions are at greatly increased risk for developing SIL. Hence the detection of HPV DNA in clinically and cytologically normal women appears to have utility in identifying women at risk for progression to SIL. This finding points to the drawbacks of using cytology alone for detecting women at risk of cervical cancer. In contrast to cervical cytology, which is highly subjective, HPV DNA testing is standardized and provides qualitative and quantitative determination of the HPV DNA present in a sample [23]. It has been proved that HPV typing has an important role in the estimation of the risk for high-grade cervical lesions [34].

In the current work, HPV DNA positivity was matching with the study of Abdel Aziz MT et al. [15] showing that the presence of SILs on cytological examination was strongly associated with HPV DNA positivity using the HC II assay. Ozturk and coworkers [1] reported that the presence of HPV together with high rates of epithelial cell abnormalities leading to cervical cancer supported the association between HPV and malignancy. This agrees with the study of Sellors and colleagues [24], who reported that the presence of SILs on cytological examination was strongly associated with HPV DNA positivity using the HC II assay. Also, Carvahalo and coworkers [35] reported that high-risk HPV types were present in every case of SIL, i.e. 100 % correlation between high-risk HPV types and cancer risk.

Among the 62 HPV positive samples, 26 HPV types were detected, whether singly (23) or in combination (38), using The LINEAR ARRAY Genotyping (LA) assay. These data were in agreement with those of Giuliani L et al. study [5], who typed 102 HPV positive samples by sequencing and by the LINEAR ARRAY Genotyping assay that showed mixed infections in 55 cases and single infections in 46 cases. Gravit PE et al. [36] showed that the PGMY09/11 primers used in the LINEAR ARRAY presented an increased type-specific sensitivity, particularly with samples infected with multiple types. This explains the increased number of multiple infections observed by the LINEAR ARRAY.

The HR-HPV positivity in this study (16.1 %) was in agreement with Panatto D et al. [33] study who recorded a prevalence of HR- HPV genotypes of 10.1 %.

Moreover, HR HPV positivity increased as our studied lesions progressed to higher grade ones. Leinonen M K et al. [37] found that none of the studied women with a high grade cervical lesion had only low risk HPV types. The same trend was observed in Argyri E et al. [31] study where HR HPV positivity was related to cytological status.

We demonstrated that LR HPV types were predominant in the cytologically normal women, while no LR types were detected in HSIL. These data were consistent with the work of Abdel Aziz MT et al. [15], where LR HPV types were detected in cytologically normal subjects, and SILs were exclusively associated with HR HPV types, while no LR HPV types were found alone in SILs.

In the study of Argyri E et al. [31] in Greece, 12.9 % of Greek women showed mixed infections, which is in harmony with our reported values (25, 6 %). In addition, the same study reported that women with cervical lesions had a higher rate of multiple infections compared to those who had normal cytology, which agrees with our findings, as all HPV types detected in cytologically normal women were single infections, while the majority of cervical lesions reported multiple HPV infection. Multiple infections might be a risk factor for development of cytological abnormalities. The majority of multi-HPV infected women harbored at least one HR HPV type.

As previously reported, LINEAR ARRAY is known to have a higher power in identifying mixed genotypes, but the clinical implication of this higher power is not well established [38–40]. One hypothesis could be that the genotyping results are due to the nature of the genotype detection used by the test: LA uses a strip that is known to be more sensitive to detect a small quantity of DNA [41].

Among the 26 HPV types detected in the 62 HPV positive women in this study, HPV 16, 18 and 31 were the most prevalent HR HPV types, and HPV 6, 62 and CP6108 were the most prevalent LR HPV. These results were consistent with data obtained from Shaltout et al. [19], who reported predominance of HPV types 16, 31, 6 and 62, with the Halfon et al. [41] results who reported HPV 16, 18 and 31 as prevalent genotypes using the same LINEAR ARRAY methodology, with the global circulating HPV types [32, 42] and with the worldwide estimates that HPV-16 and 18 are responsible for nearly 70 % of cervical cancer cases [42]. But unlike our study, Shaltout et al. [19] showed a relatively low (0.7 %) prevalence of HPV-18 in Egyptian women. Also, in contrast to our results, other studies showed that HPV 11 was the most frequent [43], while Argyri E et al. [31] found that HPV 16, 42 and 51 were the most frequent.

Both the bivalent and quadrivalent vaccines provide protection against HR HPV-16 and HPV-18 and have been reported to provide cross-protection against non-vaccine HR HPV-31, 33, 45, 52, or 58 [44, 45]. Since we found additional HR HPV types circulating in the country, cross-protective properties of these vaccines might protect Egyptian women from cervical cancers and pre-cancer lesions.

Although this was a multicentre study, conducted in 2 large hospitals, it has some limitations. First, the sample is representative of women presenting for smear testing. However, as smear taking in Egypt is not done through an organized screening system, our sample cannot be considered representative of the Egyptian population. These data may give us important information regarding regional HPV prevalence but large epidemiological studies from different regions of our country are needed. Second, whereas more than 40 HPV types exist, only 26 types were detected in this study. But all HPV types usually implicated in the origin of cervical cancer were detected. Third, the small sample size affected the significance of the results.

These data provide a complementary picture to studies of HPV type distribution in Egyptian women with and without cancer or precancerous lesions. Our results indicated a higher prevalence of HPV infection (40.8 %). They confirmed the importance of types 16 and 18 in both single and multiple infections. They provide estimates of the prevalence of other HR HPV types such as 31, and LR HPV types such as CP6108 in normal and pathological lesions, indicating that other HPV types must also be considered for further implementation of appropriate immunization and monitoring policies.

Moreover, the considerable difference in frequency of HPV types amongst previous studies evidences the need to further investigations to improve information of geographical distribution of HPV types in Egypt over time using standardized methodologies to HPV detection and typing, to apply the appropriate diagnostic and therapeutic measures, and to assess the impact of current vaccines and to guide the introduction of new generations.

## Conclusion

 HPV testing for high risk viral types using the LINEAR ARRAY assay can be used in apparently normal tissues. This test may thus facilitate the detection of silent carriers of HPV by a sensitive, non- invasive technique. It can also be used as an adjunct to cytology to provide added sensitivity. This will reduce further unnecessary investigations and could contribute to cancer prevention. Despite that LINEAR ARRAY is known to have a higher power in identifying mixed genotypes, its clinical implication needs further evaluation.
